# Intervention programs to increase influenza vaccination in Israel: which is the preferred one?

**DOI:** 10.1186/2045-4015-3-19

**Published:** 2014-05-27

**Authors:** Itamar Grotto, Rami Grefat

**Affiliations:** 1 Public Health Services, Ministry of Health, Jerusalem, Israel; 2 Department of Public Health, Faculty of Health Sciences, Ben Gurion University of the Negev, Beer Sheva, Israel; 3 Haifa District Health Office, Ministry of Health, Haifa, Israel

## Abstract

Influenza vaccine is the most effective method of preventing influenza and its complications, but coverage rates are not satisfactory. Therefore, an effective intervention is required to increase vaccination coverage. In a recent study published in IJHPR, Yamin et al. identified the need to target risk perception in the public, as a major intervention tool. Risk perception and compliance with vaccination guidelines was found to be mostly influenced by physician recommendations. These findings are in-line with similar findings in the literature, stressing the importance of patient-physician interaction in the patients’ decision to comply with vaccination guidelines produced by the public health authorities. They also underscore the need to involve primary physicians in both the decision making process as well in the vaccination campaign.

## Commentary

Influenza is an infectious disease of great importance due to several reasons, including substantial mortality, morbidity, and economic losses. Every few decades, it has struck with devastating pandemics. Moreover, Influenza is linked to incidence and mortality from one of the most prevalent non-infectious diseases - cardiovascular disease [1].

As the influenza vaccine has been found to be the most effective method of preventing influenza and its complications, many researchers have studied the determinants of one’s decision whether to get the vaccine or not, while many others have explored implementation programs (IP’s) aimed at increasing the low vaccination coverage. In an attempt to examine the combined effects of these two factors, Yamin et al. [2] conducted the study reported in a recent issue of the IJHPR.

Yamin et al. had conducted a telephone survey at the end of the influenza season aimed at a representative sample of 917 subjects in Israel. Of these, 470 (51%) fully cooperated. In addition, convenience samples of several sub-populations of particular interest (soldiers, students, health care workers, Israeli Arabs and ultra-orthodox Jews) were asked to complete paper questionnaires.

In their analysis of the survey responses, the authors divided the subjects into three vaccine acceptance categories: *Acceptors (22*%*), Conditional-Acceptors (44*%*) and Non-Acceptors (34*%*).* Acceptance was found to correlate positively with influenza risk perception and negatively with vaccine risk perception. Another Israeli research team involved in studying vaccine uptake [3,4], has likewise divided the population into three groups: compliers, trusting-reflective-non-compliers and non-compliers. Interesting, that team’s grouping did not incorporate the respondents’ risk perception levels.

Yamin et al. emphasize the need to target risk perception in the public with intervention programs primarily to increase coverage among conditional-acceptors, but also potentially to convert non-acceptors to conditional-acceptors, by raising risk perception of influenza and lowering risk perception of the vaccine. Importantly, one of the most commonly cited reasons for not getting the vaccine is fear of side effects [5,6].

In line with the literature [7], the most effective intervention program found by Yamin et al. was physician recommendation. Some authors have called for developing and evaluating interventions that help physicians and other health care professionals to more effectively implement strong and routine recommendations for vaccines. But we should not forget that the most important element that gives physicians major influence on their patient’s decision about vaccination in general and about influenza vaccination in particular, is trust. Trust is a product of a long term relationship based on respect, empathy and transparency, as seen in the case of nurses in Mother and Child Health Centers (MCHC’s) who are trusted by all, and in particular by Arab mothers in Israel. This leads to very high coverage of childhood vaccinations (unpublished data). In our opinion, the intervention program intended for physicians should start at medical schools and extend through continuous medical education for physicians. Such a program is expected to benefit both physicians and patients in various fields, including compliance with vaccination recommendations.

The second most effective intervention program was found by Yamin et al. to be information pamphlets. Firstly, the public demand for these is high, and secondly, they can be given at important and influential occasions, such as the birth of a new baby. Despite the lower efficacy of preparing and delivering pamphlets relative to physician recommendation improvement, it is much cheaper, faster and easier to implement.

Most importantly, health care professionals should not limit themselves to the one intervention program that was generally most effective, but should instead also tailor special intervention programs, or a combination of them, for special sub-populations. A recent event of polio transmission in Israel was followed by a national vaccination campaign with oral polio vaccine. The role of the primary care physicians in advocating for vaccination as well as the activities in social media via the internet, resulted in a high rate of compliance with public health guidelines [8].

Lately, the scientific community is increasingly accepting the idea that population effects of vaccines (i.e. herd immunity) are as important, if not more important, than individual effects. As mere examples, this was demonstrated in the case of vaccinating against S. pneumonia, rota virus and influenza. These population effects were achieved by vaccinating the most infectious sub-population, in case of influenza infants (above six months old) and young children [9]. This leads to the conclusion that in addition to intervention programs intended to protect individuals, efforts should be made to vaccinate the easily accessible populations of young children in schools or infants in MCHC’s. Adding the influenza vaccine to the routine vaccine schedule should be considered.

## Competing interests

The authors declare that they have no competing interests.

## Authors’ information

Prof. Itamar Grotto is the Director of the Public Health Services in the Israel Ministry of Health and an associate professor at the Ben-Gurion University of the Negev.

Dr. Rami Grefat is a Public Health Physician in the Haifa District Health Office of the Israel Ministry of Health.

## Commentary on

Yamin D, Gavious A, Davidovitch N, Pliskin J: Role of intervention programs to increase influenza vaccination in Israel. Isr J Health Policy Res 2014, 3:13.
